# An Exposition of the Natural System of the Nerves of the Human Body

**Published:** 1828-09

**Authors:** 


### REVIEWS

ART. III.—1. *An Exposition of the Natural System of the Nerves of the Human Body. With a Republication of the Papers delivered to the Royal Society, on the Subject of the Nerves. By Charles Bell, Professor of Anatomy and Surgery to the Royal College of Surgeons, &c. &c.* London. 1824.

2. *Appendix to the Papers on the Nerves, republished from the Royal Society's Transactions. By Charles Bell. Containing Consultations and Cases illustrative of the Facts announced in these Papers.* London. Longman & Co. 1827.

The Edinburgh Review for May, 1828, contains a long and elaborate article on the discoveries of Mr. Charles Bell; omitting its more declamatory parts, we present our readers with that portion which sets forth the nature and extent of those discoveries.—ED.

It is singular enough that this happy conjecture of so extraordinary a man should have been entirely overlooked, until the proofs of its truth were brought forward in Mr. Bell's experiments in 1809; for examples of diseases were daily occurring, in which sensation or motion were *separately* lost; and the mere condition of sleep showed the suspension of one set of nervous offices, and the continuance of others,—whilst the actual division of a nerve going to the extremities, took away both motion and sensation. That there were some nerves merely for sensation, and some only for motion, was, however, generally discredited, at least up to the time of Haller. If motion was lost without there being also loss of sensation, the reason was, according to Haller himself, that sensation could exist with less remaining strength than motion could! and the condition of the dying, who lost the power of moving before the power of feeling left them, was adduced as an example. This notion was allied to, f not absolutely identical with, the hypothesis of the nervous action in motion and sensation being, in kind, precisely the same, and differing only *in force*. Of those who admitted the possibility of one nerve containing distinct fibres for each of these two offices, some supposed that the conducting faculty, with relation to external and internal impressions, resided in a different substance, as that of internal impressions in the medullary, that of external impressions in the neurilemma; but many were indisposed to acknowledge the necessity for that which they conceived might be regulated in the brain or nervous centre, and for which the fibres of the nerves exhibited no visible preparation. It was urged also, that as muscular fibres could be contracted in opposite directions, so in like manner might the fibres of the same nerves convey impressions from within outwards, and from without inwards. We may here remark the curious mistake of the elder anatomists concerning the ganglions, a matter on which they affected a particular exactness, and concerning which, for the most part, they agreed. They thought the proper office of the ganglions was *to cut off* sensation from the portion of the nerve below it; it will presently be seen that the truth is diametrically opposite; and that Mr. Bell has shown all nerves of sensation to be provided with and distinguished by a ganglion.

While these obscurities continued to beset the functions of sensation and motion, there was naturally no great accord among physiologists concerning what they choose to call vitality, and animality, or concerning the distinct offices of the cerebrum and the cerebellum, or the difference between nerves of voluntary and involuntary muscular motion. The spinal marrow was considered as a simple chord, medullary without, cineritious within, giving origin to the nerves; and the curious intercostal, or sympathetic system, was sometimes asserted to be the centre of organic, nutritive, or automatic life, and sometimes regarded as hardly more than an elaborate contrivance for the propagation of sympathies among all parts of the body; between the intellect and the stomach, between the body and the mind, between one side of the body and the other, and interchangeably among all the separate organs.

Such, so vague, so obscure, so inexact, so unsatisfactory, was the kind of knowledge communicated to the student, until a very recent period; and the impression left by it was, that of confused and unintelligible profusion in the distribution of nerves, of intricacy without meaning, of an expenditure of resources without a parallel in the other works of nature. But no small part of this confusion is now made clear; what seemed to be profusion, has been shown to be a provision equally wise and economical for the perfect performance and combination of the most important and distinguishing functions of living creatures. We do not mean to speak here of the proposed location of different mental faculties in different parts of the brain, an idea which the Arabians held in their schools, and which, with many additions, has lately excited so much attention, because that theory has not yet been subjected to the kind of investigation by which its truth must finally be judged. It can hardly be said to admit of experiment, and pathological facts have scarcely yet been viewed in connexion with it. It must be confessed, indeed, that the structure and uses of the brain itself, of its eminences, cavities, divisions, and junctions, are yet among the things least understood, and still remain to be explained by some living, or some future physiologist; and many ingenious hypotheses will doubtless arise and fall, before conjecture points with any certainty to the reason of all the forms and modifications of the cerebral substance. Minute observaton of structure, and the cautious investigation of morbid phenomena, must be chiefly depended on for the elucidation of these circumstances, for here also experiment can seldom, perhaps never, be applied in aid of them. But discoveries of the highest value *have been made* in all that relates to the origins and functions of the Nerves themselves, arising from portions of the cerebrum, and from the spinal marrow; and have sprung so legitimately from anatomical investigation, have been so judiciously and so clearly illustrated by experiment, and so supported by observation of cases of disease, as to have laid a foundation, which may be enlarged or strengthened by the future progress of science, but can never be shaken or rendered insecure. Of these discoveries, so difficult in their nature, and in their practical, as well as philosophical consequences so important, the works of which the titles are prefixed to the present article, present abundant evidence, that the chief honor belongs to Mr. Charles Bell.

Previous to the publication of his Exposition of the Natural System of the Nerves, several eminent physiologists had instituted researches into different parts of this system; but the results had been less satisfactory than might have been expected from the zeal and intelligence with which they were conducted. We would by no means speak slightingly of labours most honorable to those engaged in them; yet, assuredly, it is embarrassing to those who, not being practical physiologists themselves, humbly await the discoveries of those who are, to find, after reading no small number of volumes, including the details of innumerable experiments, that by one the ganglions were looked upon as so many little brains, between which the great intercostal, or sympathetic nerve, was the chain of communication for interior life, while others considered them as cutting off the course of sensation; that by one the power of the heart, stomach, and intestines, was wholly ascribed to the spinal marrow, and by another stated to be quite independent of it; that by one the secretions were placed under the agency of the nerves, and by the experiment of another shown to be very little influenced by them.

Mr. Bell's researches have been directed more exclusively to the principles of a just and natural arrangement of the nerves, called cerebral and spinal, and to the investigation of their particular offices; and in the progress of them he has established facts, analogies and classifications, of singular value and interest.

The history of these scientific labours presents so instructive an example of the means of establishing presumed truths, and has, moreover, so much connexion with certain consequences of another kind, which we shall have to notice, that we must begin our review of what he has done for this department of anatomy and physiology, by reverting to a pamphlet circulated by him among his friends so long ago as in the year 1809, and entitled, "Idea of a new Anatomy of the Brain." In justice, it ought to be stated, that this pamphlet was never published; but was distributed among Mr. Bell's friends, for the professed purpose of eliciting objections to the course of study which he found himself then taking, and which he foresaw would, in all probability, employ the better part of his life. He wished either to be encouraged, or to be convinced that he was wrong; and notwithstanding that it contains more than the rudiments of those discoveries which we have now to explain, it seems to have excited very little attention; and twelve years of unassisted labour elapsed before Mr. Bell appeared before the public. During all that time, however, he was engaged in teaching, and each year in which he came to the discussion of nerves, was marked by improvement. An opinion then prevailed, and is probably not yet quite extinct, that the different sensations conveyed by different nerves, resulted solely from the *superior delicacy* of one set of them above the other. If the optic nerve conveyed sensations of light and colour, this was only because its terminations were, of all nervous expansions, the finest and most minute. One answer may perhaps suffice for this extravagant hypothesis. If varieties of sensation depended on gradations of nervous subtlety, any decrease in this quality would produce not the impairment of the sensation usually conveyed by the nerve so affected, but a substitution of another sense in lieu of it. If the nerve of sight was so affected, the eye would be metamorphosed into the organ of hearing or of simple touch; or in other circum stances, the sense of hearing might degenerate into that of taste. But the truth is, that the theory has no foundation in fact: for the tenuity of expansion of the nerves of hearing, or taste, or touch, has never been proved to be less delicate than that of sight itself; and however varied the impulse or agent affecting a nerve, the impression always takes its character from that of the nerve itself. Mr. Bell, however, soon applied himself to the correction of more formidable errors: and attacking the common opinion, that a separate sensation and volition are conveyed by the same nerves, he asserted the functions of different parts of the cerebrum and cerebellum, and maintained that a great part of the nerves were not single nerves possessing various powers, but bundles of different nerves, the filaments of which were united for the convenience of distribution, but yet as distinct in their office as in their origin; that the perception or idea depended on the part of the brain to which the nerve was attached; and that the matter of the nerves of the external organs of sense was adapted to the reception of certain impressions only: further, "That the nerves of sense, the nerves of motion, and the vital nerves, are distinct through their whole course, though they seem sometimes united in one bundle; and that they depend for their attributes on the organs of the brain, to which they are severally attached."

We find Mr. Bell strongly insisting, in this pamphlet, on the complete distinctness of the cerebrum and cerebellum; and pointing out the distinction, (which afterwards led him to other distinctions of great moment,) that although the two hemispheres of the brain were so like in form and substance, and so united by tracts of medullary matter, as to make consentaneousness of office both probable and easy, the cerebrum and cerebellum were, on the contrary, very different both in form and arrangement, and but slightly and indirectly connected; adducing facts at the same time which showed that these parts might be separately affected; and, finally, deducing from these observations the conclusion of their being distinct in office. This conclusion was strengthened by reference to the varying proportions of the cerebrum and cerebellum in different classes of animals, the diversity of the former in creatures differently endowed, and the general permanency of character in the latter. It is of much consequence, as will hereafter be evident, to quote in this place Mr. Bell's own description of his attempts to prove by experiment what he had suceeeded in making so probable by reasoning:—

"I took this view of the subject. The *medulla spinalis* has a central division, and also a distinction into anterior and posterior fasciculi, corresponding with the anterior and posterior portions of the brain. Further, we can trace down the crura of the *cerebrum* into the anterior fasciculus of the spinal marrow, and the crura of the *cerebellum* into the posterior fasciculus. I thought that here I might have an opportunity of touching the *cerebellum*, as it were, through the posterior portion of the spinal marrow, and the cerebrum by the anterior portion. To this end I made experiments, which, though they were not conclusive, encouraged me in the view I had taken.

'I found that injury done to the *anterior* portion of the spinal marrow convulsed the animal more certainly than injury done to the posterior portion; but I found it difficult to make the experiment, without injuring both portions.

'Next, considering that the spinal nerves have a *double* root, and being of opinion that the properties of the nerves are derived from their connexions with the parts of the brain, I thought that I had an opportunity of putting my opinion to the test of experiment, and of proving at the same time, that nerves of different endowments were in the same cord, and held together by the same sheath.

'On laying bare the roots of the spinal nerves, I found that I could cut across the posterior fasciculus of nerves, which took its origin from the posterior portion of the spinal marrow, without convulsing the muscles of the back; but that on touching the anterior fasciculus with the point of the knife, the muscles of the back were immediately convulsed.

'Such were my reasons for concluding that the cerebrum and the cerebellum were parts distinct in function, and that every nerve possessing a double function, obtained that by having a double root. I now saw the meaning of the double connexion of the nerves with the spinal marrow; and also the cause of that seeming intricacy in the connexions of the nerves throughout their course, which were not double at their origins.

'The spinal nerves being double, and having their roots in the spinal marrow, of which a portion comes from the cerebrum, and a portion from the cerebellum, they convey the attributes of both grand divisions of the brain to every part; and therefore the distribution of such nerves is simple, one nerve supplying its destined part. But the nerves which come directly from the brain, come from parts of the brain which vary in operation; and in order to bestow different qualities on the parts to which the nerves are distributed, two or more nerves must be united in their course or at their final destination.—Hence it is, that the first nerve must have branches of the fifth united with it: hence the *portio dura* of the seventh pervades everywhere the bones of the cranium to unite with the extended branches of the fifth: hence the union of the third and fifth in the orbit: hence the ninth and fifth are both sent to the tongue: hence it is, in short, that no part is sufficiently supplied by one single nerve, unless that nerve be a nerve of the spinal marrow, and have a double root, a connexion (however remotely) with both the cerebrum and cerebellum.'

In this passage we see laid open the very foundation of all Mr. Bell's important discoveries relating to the functions of the nerves: it being perfectly clear that he was then aware of the distinct offices of the anterior and posterior portions of the spinal marrow; and that the nerves arising from its anterior part were for Motion, and those of the posterior were for Sensation, or for some other office; for there was much difficulty in proving the latter circumstance by direct experiments, in consequence of the general shock communicated; but the fact was afterwards proved by experiments made on the functions of the fifth nerve. It is no less clear that Mr. Bell then understood that the nerves proceeding from the brain had each but a single function, and required to be united with some other nerve when a double function was to be effected: and although it was in consequence of these investigations, and from the discovery of this general principle of combination of separate nerves for combined offices, that he was led to the understanding of the extensive system of nerves connected with the complicated actions of respiration; yet this is precisely the part of Mr. Bell's labours, concerning which justice has been most withheld from him. His incontestable discovery of the distinct functions of the spinal nerves has been claimed by others; and those who seem most disposed to pronounce fairly on this matter in another country, where the claim has been most directly advanced, and would at least allow Mr. Bell a share of the merit of priority, rest his title to it, curiously enough, on the *probability* of his having been naturally led to the discovery *by* his researches into the system of the respiratory nerves; whereas the truth is, that these researches, with all their consequences, were *the result* and not *the cause*, of his having discovered the principle on which the spinal nerves of motion and sensation were distributed. It was, beyond all question, by the light of this principle that he was directed to the conclusion, that the nerves of an organ were complex only because its functions were complicated. Putting aside the mere evidence of dates, there is proof enough of Mr. Bell being the first discoverer, from the course of his progress. We see, first, the observation of structure, leading him to infer the distinct functions of the different roots of the spinal nerves; and then the inconvenience of the experiments directly proving it, causing him to trace analogical double structure in some of the cerebral nerves, and to select the fifth, of which he succeeded in showing the double office. Subsequently to this, we observe him still seeing more nerves sent to parts already supplied with nerves both of motion and sensation, and led to investigate their uses; and finally, by experiment, by comparative anatomy, and pathological phenomena, ascertaining the separate and *superadded* system of respiratory nerves, so denominated by him with an obvious reference to the previously ascertained nature of the *regular* nerves of sensation and of motion. Every step of this process is plain and orderly, and it would be difficult to find a more admirable example of useful discovery philosophically attained. It is enough, for the present, to have pointed out the singular perversity of fortune by which Mr. Bell's persevering and patient pursuit of a great principle, and his meritorious abstinence from premature assertion, had almost deprived him of the credit which so truly belongs to him.

Although, from circumstances, it has become necessary to trace Mr. Bell's discoveries back to this pamphlet, it is proper to remark, that the observations on the brain and the roots of the nerves, were not the commencement of his labours. It was the seeming *complication* in the course of the nerves, as they wander over the head, the neck, and the chest, that first led him to this investigation.—When he was directing our attention to the columns of the spinal marrow, and the double roots of the nerves, he was in fact explaining that intricacy, which he found in the distribution of the nerves, and which had met him each time he returned to the demonstration of the subject in his public lectures. So that he has, in fact, arrived at his conclusions by two different paths,—1st, by observing the distribution and relation of the nerves in their remote extremities; and, 2d, by observing the columns of the brain and spinal marrow, and the origins of the nerves from these columns.

Before following the order of Mr. Bell's papers, it may facilitate the comprehension of the whole subject to state, as he himself has done in his preliminary observations, that according to the views he takes of the nerves of the human body, there are, besides the nerves of vision, smell, and hearing, *four* different systems of nerves distributed through the body; those, namely, of Sensation, of Voluntary Motion, of Respiratory Motion, and those which, neither communicating sensation in the ordinary meaning of that term, nor conveying the volition which directs voluntary motion, nor yet respiratory, unite the body into a whole, and are essential to Nutrition, to growth, to decay, and generally to animal existence. The nerves of these separate functions are undistinguishable by their structure, but known by their origins, they are sometimes separate in their course, sometimes bound together in one sheath; but never, as had been formerly supposed, confounded in office. They are divided into simple nerves and compound; the first having their roots arising in a line, or sequence, from the brain or spinal marrow, as seen in the ninth nerve; the roots of the second arising in *double* rows, each row from a *different* column or tract of nervous matter, as exemplified in the nerves arising from the spinal marrow.

'If we were successfully to trace a nervous cord, (we shall suppose from a muscle of the fore-arm,) it would be found a simple filament, thread, or funiculus. We should then trace it into a oompound nerve; perhaps the ulnar nerve, which we call compound, because there are in it filaments of motion, and filaments of sensation bound together. At the root of the auxiliary nerve we should trace it into the composition of a fascis, where it forms the anterior root of a spinal nerve. Being further traced, it would merge in the anterior column of the spinal marrow; and traced into the base of the brain, it might be followed as a *tractus*, a streak of matter distinguishable from the surrounding substance, until it was seen to disperse and lose itself in the cineritious matter of the cerebrum. In all this extent, however combined or bound up, it constitutes one organ, and ministers to one function, the direction of the activity of a muscle of the hand or finger. Even in this respect its operation is not perfectly simple, for while it excites the muscle to change its state, which we call its state of contraction or of relaxation, it also conveys to the sensorium a sense of the condition of that muscle.

'And so, if we trace other fasciculi, or rather filaments, whether they be for the purpose of sensation or of motion, each retains its office from one extremity to the other; nor is there any communication betwixt them, or any interchange of powers, further than that a minute filament may be found combined with filaments of a different kind, affording a new property to the nerve thus constituted.'

Mr. Bell describes the spinal marrow as being composed in reality of six columns, or three in each lateral portion; an *anterior* column, which is for the function of Voluntary Motion, and may be traced into the substance of the brain; a *posterior* column for Sensation, and a third, or lateral, column, between the anterior and posterior columns, and which is for the Respiratory functions. The existence of this last had been pointed out by other anatomists; but Mr. Bell was the first clearly to describe it, or to point out its peculiar use. Each of these columns has subdivisions, not yet explained. This arrangement of the spinal marrow prevails in all the vertebrated animals; and is indispensable to the association and combination of all the movements connected with the act of respiration in those possessing a thorax capable of respiratory motions.

To speak first of the *regular* nerves, there are of these thirty sent out from the spinal marrow on each side; and each of these has two distinct roots, one from the anterior and one from the posterior column. The *posterior* root of each is distinguished by a ganglion, situated where it is surrounded by the sheath of the spinal marrow, and before its junction with the anterior root. Seeing this great regularity of the spinal nerves, and the very great irregularity of the cerebral nerves, Mr. Bell was led to inquire into the reason of so remarkable a contrast; whether, first, the double office of these nerves depended on their having double roots; and whether this was the cause of their peculiar simplicity of arrangement: and, secondly, what cerebral nerves, in their distribution to the head and face, had similar offices.

'It was necessary to know, in the first place, whether the phenomena exhibited on injuring the *separate* roots of the spinal nerves correspoded with what was suggested by their anatomy. After delaying long on account of the unpleasant nature of the operation, I opened the spinal canal of a rabbit, and cut the posterior roots of the nerves of the lower extremity; the creature crawled, but I was deterred from repeating the experiment by the protracted cruelty of the dissection. I reflected, that an experiment would be satisfactory, if done on an animal recently knocked down and insensible; that whilst I experimented on a living animal, there might be a trembling or action exerted in the muscles by touching a sensitive nerve, which motion it would be difficult to distinguish from that produced more immediately through the influence of the motor nerves. I, therefore, struck a rabbit behind the ear, so as to deprive it of sensibility by the concussion, and then exposed the spinal marrow. On irritating the posterior roots of the nerve, I could perceive no motion consequent, on any part of the muscular frame; but on irritating the anterior roots of the nerve, at each touch of the forceps there was a corresponding motion of the muscles to which the nerve was distributed. These experiments satisfied me that the different roots and different columns from whence those roots arose, were devoted to distinct offices, and that the notions drawn from the anatomy were correct.

'The anterior roots of the spinal nerves, and the anterior column of the spinal marrow, being thus shown to have a power over the muscular system, the next step of the inquiry was distinctly indicated. If I pursue the tract of the anterior column of the spinal marrow up into the brain, shall I find the nerves which arise from it to be muscular nerves? An anatomist will at once answer, that only muscular nerves arise in this line.'

Mr. Bell's descriptions are materially aided by drawings and plans; but those who are acquainted with anatomy will remember, that on tracing up the anterior column of the spinal marrow into the corpus pyramidale, the ninth nerve is found to arise from it, having *one* series of roots only, corresponding with the anterior roots of the spinal nerves, and that this nerve is a nerve of Motion, entirely devoted to the muscles of the tongue, and unconnected with the sense of taste. Higher up, arising from the same *tractus motorius*, is the sixth nerve, a muscular nerve of the eye; and higher still, tracing the tractus through the Pons Varolii, are the roots of the third nerve, the motor nerve of the eye. These interesting points being ascertained, it remained to be seen whether the posterior column of the spinal marrow, and the roots proceeding from it, were for sensation. Pursuing this inquiry,it was found that the fifth nerve the sole nerve of sensation in the head and face; and it was thence understood why there was no necessity for the third, sixth, and ninth nerves having a posterior or ganglionic root. Thus far the fifth nerve agreed with the spinal nerves in bestowing sensation: It then became a question whether it had a further resemblance to them, and comparisons being instituted, both in man and in brutes, of the anatomy of this nerve and of the spinal nerves, a remarkable similarity was discovered. It was seen, like the spinal nerves, to have *a double root*, the anterior root passing the ganglion, and the posterior falling into the ganglion; and on following back the anterior root, it was observed to come out between the *funes* of the pons varolii, and, in fact, from the crus of the cerebrum. And as the anterior portion of the nerve did not enter the ganglion, Mr. Bell conceived it to be, in fact, the uppermost nerve of the spine, the uppermost of that series of nerves which are both for motion and for sensation. To ascertain the correctness of this conclusion, the nerve was exposed at its root, in an ass just killed, and being irritated, 'the muscles of the jaw acted, and the jaw closed with a snap.' The nerve was next divided in a living animal, and the jaw was found to fall.—These experiments left the functions of the nerve no longer a matter of doubt, and proved it to be both a muscular nerve and a nerve of sensibility; in short, to be, for the head, what the spinal nerves are for the other parts of the body.

The *regular* nerves, then, are the seven cervical, the twelve dorsal, the five lumbar, the six sacral, the sub-occipital, and the fifth pair, and all these nerves, which are for sensation and motion, are *double* in their origin; and they are common to all animals, 'from the worm up to man.' The *irregular* nerves, so designated from the irregularity of their distribution, have *a single* root, and are superadded to those just spoken of, according to the complication of organs for which they are intended; such are the third, fourth, and sixth nerves going to the eye; the seventh, to the face; the ninth, to the tongue; the glossopharyngal, to the pharynx; the nervus vagus, to the larynx, heart, lungs, and stomach; the phrenic, to the diaphragm; the spinal acessory, to the muscles of the shoulder; and what Mr. Bell has named the External Respiratory, a nerve resembling the phrenic of its origin, but sent to the outside of the chest, to the serratus magnus muscle, which muscle is also supplied from the regular system of nerves All these irregular nerves are, we have said, using Mr. Bell's words, *superadded*, and for superadded organs; and this superaddition of organs he has shown to be such as are connected with the apparatus of respiration, and the variety of offices for which this apparatus is prepared in the higher animals. It is this explanation which gives clearness to a piece of anatomy, formerly of all the most difficult and confused, and which enables us to account for the apparent prodigality, for the countless ramifications, for the numerous connexions of the nerves of the face, neck, and chest; more particularly of the fifth and seventh nerves with the ninth, and with the cervical and phrenic nerves.

The motions connected with respiration, although chiefly seen in the neck, trunk, and face, extend almost all over the body, to some hundred muscles, as is evident during any of the more violent respiratory efforts. Here, then, are new motions, differing wholly from those of which the end is locomotion, and no less from the voluntary motions, and therefore presumably requiring a new source of nervous energy. Of these motions, some are well known to be under the command of the will, others carried on independently of the will, and not to be controlled for any long time by the strongest effort of volition, but still carried on by the same muscles, both when we will, and when we do not. Thus we breathe during sleep without volition, and yet we can use the respiratory muscles as voluntary muscles in violent exertions, or in singing. Hence there is an evident necessity for *a combination* of powers; and from this combination arises a necessary degree of complexity in the system of nerves connected with those powers. Mr. Bell has illustrated this very clearly:—

'Let us observe, in the act of eating and swallowing, the necessary combination of the three powers of sensation, voluntary muscular activity, and the act of the respiratory muscles. If we cut the division of the fifth nerve which goes to the lips of an ass, we deprive the lips of sensibility; so when the animal presses the lips to the ground, and against the oats lying there, it does not feel them; and consequently there is no effort made to gather them. If, on the other hand, we cut the seventh nerve where it goes to the lips, the animal feels the oats, but it can make no effort to gather them, the power of muscular motion being cut off by the division of the nerve. Thus we perceive that in feeding, just as in gathering any thing with the hand, the feeling directs the effort, and two properties of the nervous system are necessary to a very simple action.

'In drinking, the fluid is sucked in by the breath, and when the mouth is full we swallow. The water is felt; the lips are moulded into the right form by volition, and the muscles of inspiration combine to draw in the fluid. In the act of swallowing, the liquid would descend into the windpipe, were there not a combination of the muscles of respiration with the apparatus of deglutition to prevent it; nor could the fluid or the solid morsel pass the diaphragm without a similar coincidence of activity and relaxation betwixt parts animated by different systems of nerves.

'In speaking, it is still more obvious that the act of respiration must become voluntary, in order to push out the breath in combination with the contractions of the larynx, and tongue and lips, for producing sound, and more especially articulate language.

'The respiratory system must be exercised under an instinctive and involuntary impulse, as in breathing during sleep and insensibility. But it must, at certain times, be associated into voluntary actions. By foreseeing this difficulty, we shall avoid the danger of pushing the investigation of the anatomy too far, or of throwing a doubt over important discoveries by attempting too much.'

It was fortunate for Mr. Bell's investigations, that the face was a part of the body in which the nerves of voluntary motion and of sensation, which in other parts of the frame ran their course bound up together, were distinctly and separately distributed, coming out from the cranium through separate openings, and meeting only in their terminations. This arrangement enabled him to make those decisive experiments to which we have already alluded.

We have before said that our author had contrasted the spinal nerves, which are remarkable for their regularity, with the nerves of the brain, which are irregular both in their origin and course. The former arise uniformly from prolonged tracts of medullary matter, and have each *two* roots and a ganglion upon one, and they are distributed universally over the body. The nerves of the brain arise from distinct parts, and their roots are *single*, with the exception of one, which is the fifth. We find that this has *two* distinct origins, and also a ganglion upon one of them—that it corresponds, in fact, with the extensive class of spinal nerves. Here there is a remarkable circumstance demanding explanation: when we find a spinal nerve introduced in the midst of so many others, it points out distinctly that the spinal nerves must have some peculiarity of function. The fifth nerve was found, accordingly, to be both a nerve of motion and of sensibility, as all the spinal nerves are. But the question then arose, what necessity is there for another nerve of motion being supplied to the muscles of the face, where the fifth is already distributed? This can only be answered by attending to the peculiar functions performed by these muscles, and observing how they are connected with the complex actions of respiration, as may be seen in simple breathing, or speaking, and expression. It would thus appear that the fifth pair is not suited for controlling these actions, but that a new nerve having a different origin from the brain is required. All these circumstances being considered, Mr. Bell was prepared to decide by experiment whether the fifth and seventh afforded a double supply of the same kind of endowment, or performed different offices.

'An ass being thrown, and its nostrils confined for a few seconds, so as to make it pant and forcibly dilate the nostrils at each inspiration, the portio dura was divided on one side of the head; the motion of the nostril of the same side instantly ceased, while the other nostril continued to expand and contract in unison with the motions of the chest.

'On the division of the nerve, the animal gave no sign of pain; there was no struggle nor effort made when it was cut across.

'The animal being untied, and corn and hay given to him, he ate without the slightest impediment.

'An ass being tied and thrown, the superior maxillary branch of the fifth nerve was exposed. Touching this nerve gave acute pain. It was divided, but no change took place in the motion of the nostril; the cartilages continued to expand regularly in time with the other parts which combine in the act of respiration; but the side of the lip was observed to hang low, and it was dragged to the other side. The same branch of the fifth was divided on the opposite side, and the animal let loose. He could no longer pick up his corn; the power of elevating and projecting the lip, as in gathering food, appeared lost. To open the lips the animal pressed the mouth against the ground, and at length licked the oats from the ground with his tongue. The loss of motion of the lips, in eating was so obvious, that it was thought a useless cruelty to cut the other branches of the fifth.

'The experiment of cutting the respiratory nerve of the face, or *portio dura*, gave so little pain, that it was several times repeated on the ass and dog, and uniformly with the same effect. The side of the face remained at rest and placid, during the highest excitement of the other parts of the respiratory organs.

'When the ass, on which the respiratory nerve of the face had been cut, was killed, which was done by bleeding, an unexpected opportunity was offered of ascertaining its influence, by the negation of its powers on the side of the face where it was cut across.

'When an animal becomes insensible from loss of blood, the impression at the heart extends its influence in violent convulsions over all the muscles of respiration; not only is the air drawn into the chest with sudden and powerful effort, but at the same instant the muscles of the mouth, nostrils, and eyelids, and all the side of the face, are in a violent state of spasm. In the ass, where the respiratory nerve of the face had been cut, the most remarkable contrast was exhibited in the two sides of its face; for whilst the one side was in universal and powerful contraction, the other, where the nerve was divided, remained quite placid.'

'The actions of sneezing and coughing are entirely confined to the influence of the respiratory nerves. When carbonate of ammonia was put to the nostrils of the ass whose respiratory nerve had been cut, that side of the nose and face where the nerves were entire, was curled up with the peculiar expression of sneezing; but on the other side, where the nerve was divided, the face remained quite relaxed, although the branches of the fifth pair and the sympathetic were entire. The respiratory nerve of one side of the face of a dog being cut, the same effect was produced; the action of sneezing was entirely confined to one side of the face.

'On cutting the respiratory nerve on one side of the face of a monkey, the very peculiar activity of his features on that side ceased altogether. The timid motions of his eyelids and eyebrows were lost, and he could not wink on that side; and his lips were drawn to the other side, like a paralytic drunkard, whenever he showed his teeth in rage.'

'All that excitement seen in a dog's head, his eyes, his ears, when fighting, disappears, if this nerve be cut. The respiratory nerve being cut across in a terrier, the side of the face was deprived of all expression, whether he was made to crouch, or to face an opponent and snarl. When another dog was brought near, and he began to snarl and expose his teeth, the face, which was balanced before, became twisted to one side, to that side where the nerve was entire; and the eyelids being, in this state of excitement, very differently affected, presented a sinister and ludicrous expression.'

About a year after the communication of these facts to the Royal Society, Mr. Bell read a second paper in continuation of the same subject, but relating particularly to the nerves which associate the muscles of the *chest* in the actions of breathing, speaking, and expression. The extensive combinations into which these muscles enter may be conceived, by reflecting on the actions and offices of this portion of the body, which supports and protects the heart and lungs, as well as the viscera seated in the upper part of the abdomen, and produces, by alternate opposition and yielding to the atmospheric pressure, the action of respiration. In order to explain these actions, Mr. Bell found it necessary to draw attention to the muscles of the chest; observing particularly upon those which come from the shoulder bones, and from the head, and descend to the upper part of the chest, and in explaining how these muscles expand the chest, as in drawing breath, he proves that at the same moment the head must be raised, and the shoulders drawn back. Having thus shown by the review of the muscles, which of those on the fore part and of those on the back part of the chest, are necessarily combined in the act of drawing breath, he has paved the way for removing the whole intricacy of this part of the Nervous system: for to reach these respiratory muscles, the raspiratory nerves must take a devious course, turning and twisting, and threading through the nerves of sensation and voluntary motion. Taking one of these nerves, the spinal accessory, and dividing it, he found that the muscles supplied by it were cut off from partaking in the act of breathing, whilst they retained their office under the other nerves; that is, could be used as voluntary muscles, when they no longer acted as respiratory muscles. To the spinal accessory nerve, therefore, Mr. Bell has given the name of Superior Respiratory; and he has ascertained that whilst the phrenic acts on the diaphragm in expanding the chest, the external muscles associated with the diaphragm are combined in such action by a similar provision of respiratory nerves; that what the phrenic, or internal respiratory, is to the diaphragm, the spinal accessory is to the muscles behind the neck and to the mastoidens, and the external thoracic to the muscles of the sides of the chest. The proofs of these opinions rest, like those of the office of the respiratory nerve of the face, on the origin and distribution of these nerves in the human subject, on the facts exhibited by comparative anatomy, or pathological phenomena, and on experiment. It has been seen that the spinal accessory arises, like the other respiratory nerves, from the lateral column of the spinal marrow. In fishes, which have no diaphragm, there are no phrenic nerves; nor are there any spinal accessory, or external thoracic, their muscular conformation not requiring any. The structure of the wing, and the absence of a mastoid muscle, render a spinal accessory nerve unnecessary to birds. Quadrupeds in general have all the three respiratory nerves of the trunk; but the camel, which is without a mastoid muscle, its neck and head being supported by a succession of muscles which are shorter and attached to the vertebræ, has no spinal accessory nerve.

By inquiring into the functions of the numerous distinct nerves which go into the parts surrounding the eye, Mr. Bell was led to observe the motions of the eyeball, with particular interest. The motions of the eye, consequently, became the subject of one of his papers, the third, read before the Royal Society, in which it was explained why no less than six nerves were sent to that organ, and crowded into a space so small as that of the orbit. The internal arrangements of this important organ of sense, have often furnished a pleasing subject of proper illustration, and are pretty generally understood; but what Mr. Bell terms 'the frame-work which suspends it, and by which it is covered and protected,' has been less attended to, though no less worthy of notice than the contrivances within the eye itself. Except when some part of this curious external apparatus is impaired, we seldom are aware of its great value. Paley was struck with the importance of the 'two little muscles that serve to lift up the eyelids,' by being acquainted with a gentleman who had lost the use of them, and was obliged 'to shove up his eyelids with his hands;' and there are other minute offices without the regular performance of which sight would be equally interfered with. Mr. Bell considers the motions of the eye in two points of view; with relation to mere vision, and with relation to the preservation of the organ. He has pointed out a peculiar revolving motion which had not previously attracted attention, and that the different conditions of the retina are accompanied by appropriate conditions of the surrounding muscles; he has divided these muscles into two classes, one presiding over the organ when we use it, the other taking charge of it when we sleep, or during faintness and insensibility; and he has shown the deductions which may be drawn from these circumstances, in connexion with the appearances of the eye in disease, and as an organ of expression.

The revolving movement of the eye is that by which, when the eyelids are closed, the cornea, or transparent part, is raised under the upper lid, a movement easily verified by closing one eye and placing the finger over the eye-lid, so as to feel the ball of that eye, and then shutting the other eye, in which case the globe of the eye over which the finger is placed is felt to move upwards, as it is also perceived to descend when the other eye is opened again. The intention of this motion is thus explained by Mr. Bell. The margin of the eyelids is flat, and when the lids are closed, they meet only by the outer edge of this flat margin, and a gutter is left between them and the cornea; this part of the cornea, therefore, in the space or gutter so left, would never be touched or swept over by the eyelids, unless the eyeball shifted its place in the act of shutting the eye; and it would consequently become dimmed by a continual collection and accumulation of moisture there, exactly in the axis of the eye. This motion of the eye also facilitates the flow of tears from the lachrymal duct, and is performed so rapidly, the globe of the eye moving upwards whilst the lid moves downwards, that protection is instantaneously given in all emergencies to which so delicate an organ is exposed, and which its outward guards are not of themselves calculated to meet. The best illustration of this motion of the eye, and one which Mr. Bell has often pointed out to his pupils in the Middlesex Hospital, is afforded in some cases of partial paralysis; cases in which there is no loss of sensation, but in which the motion of the eyelids of one eye is lost, and the eyelids remain open; in these instances, whenever the unaffected eye is closed, whenever the patient winks, the eyelid of the paralyzed side of the face is unmoved, but the globe of the eye is at that moment lifted upwards, as if to get under the raised lid; and this circumstance, it may be observed, not only shows the revolving motion of the eye, but the dependence of that motion on a nerve not involved in the paralytic affection which deforms the face. Mr. Bell further observes, that during this rapid motion, the lower eyelids move also, and in a direction towards the nose; so that if both lids are marked, it may be seen, that, when the eye is shut and opened, the spot on the upper lid descends, and ascends perpendicularly, while that on the lower moves horizontally towards the nose and from it, 'like a shuttle.' So that we see the whole effect of the act of winking, an operation so instantaneous as to have formed the basis of a proverb from time immemorial, is, that the secretion of the lachrymal gland is promoted, and directed towards the duct along which it is to flow from the eye after washing it, the upper eyelid descends and sweeps the eye; the globe of the eye is so moved, that the only part which could not be so swept is effectually cleared; and the under eyelid moves, so as to propel towards the outlet of the eye, at its inner angle, whatever has offended it, whatever would obscure or impair it, as well as the tears which have been used to wash these objects away. At the same time, the eye is relieved by the interchanges of muscular action which take place during this momentary action, and it is immediately protected when immediate protection is required.

These are but a small part of the proofs of design afforded by this single organ, contributing as it does so largely to the knowledge and the happiness of living creatures. In an examination of the works of Infinite power, we are always reminded of the benevolence with which it is combined, and of the limitation of our own conceptions. The larger works of the creation first attract our regard; we push our ambitious enquiries into the heavens, and are lost in ideas of space and magnitude, of rapidity and immensity of movement beyond the reach even of imagination. But if we look down into the smallest details of those works which are around us, or which are exhibited in the 'express and admirable' structure of our own frame, we are no less delighted by exquisite contrivance and delicacy of execution. The more we increase our means of acquiring a knowledge of the larger features of the universe, the more we are impressed with the greatness of the power which sustains and directs so stupendous a work; and the more minutely we pursue researches which our unassisted senses could not institute, the more delicate, the more immeasurably superior to any performance of human hands, do all the divisions and parts of the vast work appear.

Nothing, we think, can be more fortunate than the induction by which Mr. Bell has thus unfolded the marvellous contrivances that are combined in the arrangement of the eye—having demonstrated with admirable patience and precision, that there are five different objects to be provided for by appropriate nerves:—1. vision; 2. common sensibility; 3. the voluntary direction of the eye-ball; 4. the condition of the eye in sleep, or at perfect rest; 5. the instinctive and rapid motions in winking, to protect the eye whilst it is exercised and watchful; and lastly, it has been shown, by some curious observations, in a manner quite unexpected, that there is a necessity for a connexion between the muscles of the eye, and the muscles of respiration generally, without which this delicate object would suffer derangement. It was only by such minute attention to the functions of this part that he could arrive at the rationale of the number of nerves (no less than six) going into the orbit.

Our author has certainly grappled here with the most difficult part of the whole system: And yet the advantage of his method might perhaps have been better illustrated by attending to the different operations of the tongue, which is the instrument of deglutition—the principal instrument of articulate language—the seat of an exquisite sense of touch—and the organ of taste. This explains why there are three nerves distributed to it, each of which forms different connexions with other nerves; that nerve which is the organ of taste being connected with the salivary glands and muscles of mastication; that which connects the muscles of the tongue with those of the fauces in swallowing, passes down to the muscles of the pharynx; whilst that which is the organ of volition is connected with the nerves of the larynx, and the nerves of respiration generally,—to combine the actions of the tongue in articulate language with the act of breathing.

It has too commonly happened that those who have been ambitious to improve medical science have neglected this instructive study, for the sake of experimenting. The example of reserved and judicious appeal to experiment, in support of views derived from attention to structure, presented in these labours of Mr. Bell, is, in this point of view, particularly instructive. Whilst he never seems to have performed an experiment which could have been avoided, and has decidedly escaped all reasonable censure on the score of cruelty, not one of his experiments appears to have been idle or fruitless. The rash and unadvised destruction of animal life, in quest of whatever may chance to turn up, or of whatever is new and rare, can by no pretext be justified. The subject which now occupies us has been followed with every variety of investigation, and attempts have been made to snatch, as it were, a knowledge of the most hidden things of the Nervous System, by experiments without number; yet the results of injuries, most heedlessly inflicted on various portions of the cerebral mass cannot be said to have led to conclusions that we can at all rely upon, even respecting the parts actually involved in such injuries. In many instances the only effect has been the loss of power to direct the movements of the body, and a general diminution of sensorial power—from which no precise conclusion can be drawn. All that has been got at in this way, with all the diligence and all the disregard of life, by which the attempts have been characterised, is not to be compared with the valuable results obtained by those physiologists who, labouring more patiently, and having caught a steady view of some important fact, have first mutually considered which, of many paths, would most surely lead them to the point; and who have then, by careful reasoning, aided by knowledge industriously gathered from various sources, proceeded, by the light of a few decisive experiments, step by step, to what has been hailed, by all competent judges, as equally valuable and indisputable. Such physiologists proceed like the skilful miner, who, having first ascertained, from indications that cannot err, that there is a precious vein beneath the surface, commences his labour when it can be commenced with the most advantage.—Those of another description are like wild speculators, the Dousterswivels of science, who either proclaim a treasure when none exists, or lose their time in indiscriminate search; and, in their eagerness for gold,assert what is not true, or waste their illdirected industry on hopeless and ungrateful soils. We are told by Celsus that, even before his time, and in the age of Alexander, Erasistratus indulged himself very freely in the examination of the bodies of criminals whilst they were yet alive, 'etiamnum spiritu remanente,' and saw the contractions of the heart, and beheld the lacteal vessels of the mesentery filled with chyle; but it was many hundred years later before the use of the lacteals, or of the action of the heart, were comprehended; and the knowledge of their uses was gained in a very different manner. The immortal Harvey, in relating his own progress, says, '*I began to think whether there could be* any motion of the blood, as in a circle; *which I afterwards found to be true.*'—And thus it has probably always been, and must always be, with great discoveries. In the midst of patient research, accident presents a single fact unthought of before, and the fact kindles a train of conjecture; a possibility is imagined; the faculties employed in the investigation and comparison of facts, are called into exercise; reasoning suggests experiment, and directs pursuit; careful observation governs and regulates the whole process, and a great truth is evolved.

It would be to forget half the value of the enlightened views of the structure and functions of the Nervous System which have occupied our attention, if we were not to allude to the great improvement that may be expected to arise from them to medical science. From understanding the anatomy and office of any part of the system, to clear ideas of the diseases of such structures of functions, the step is easy and direct; and when both are understood, it is not difficult to apply principles of treatment with increased effect. To the Surgeon there are many very obvious applications of the information given by Mr. Bell, concerning the properties of two great nerves of the face. Here the division of different nervous branches has been frequently performed for the relief of that distressing disorder, the Tic Doloureux; sometimes with complete success, but sometimes not only without benefit, but with consequences highly inconvenient; events which can now be explained. Whether the branches of the portio dura, or respiratory nerve of the face, may not in certain states of disease be affected with pain, is perhaps a question not quite decided; but it is easily understood, from the uses of this nerve, that if any attempt is made to relieve pain by dividing any part of it, there will be a loss of motion in some part of the muscles of the face, and consequent deformity; and if in this, or any other operation about the eye-lids, this nerve should be divided in that situation, consequences still more unfortunate would ensue; for it is the nerve on which the motions of the eyelids depend, and as they could not then be closed, the cornea of the undefended eye would become opaque, and the sight of the eye would be lost.

When a man has been deprived of all sensation and power of voluntary motion below the lower part of the neck, by an injury of the spine in that part, but continues to inspire and expire naturally, can cough, blow his nose, &c., we learn from Mr. Bell's observations, that the continuance of his respiration does not depend on the continued power of the phrenic nerve alone, of which the origins are higher than the seat of the injury; and we comprehend, that if the motions of the thorax and abdomen depended wholly on the regular nerves, we should see *them* also suspended. But the phenomenon is sufficiently explained, when we remember that these alternate respiratory motions are a consequence of the unimpaired state of the respiratory nerves in a wider sense, of the phrenic for one certainly, but also of the superior and external respiratory nerves, of which the origins are as high as those of the phrenic. If the injury happens above the orign of the phrenic nerve, but not so high as that of the spinal accessory, breathing will still be carried on, though for a short time only; the action of the diaphragm is lost, and the patient breathes with much effort of the shoulders, between which the head is almost drawn at every inspiration; those muscles being now principally called into action which are supplied by the spinal accessory. In these, and other accidents affecting the spinal column, the surgeon is therefore now enabled to judge more correctly of the seat of the injury, often inaccurately referred to by the patients themselves; and he is enabled better to assign a reason for the continuance of life in deep and serious wounds of so important an organ as the brain; as well as to account for the strange circumstance which is witnessed in some monstrous births, in which respiration is performed, although there is no brain at all.

To the physician, the advantages of an exact acquaintance with the Nervous System are even greater. Instead of rejecting nervous theories, as some eminent practitioners have done, in the most unqualified terms, the physician may now find in this delicate system secure foundations for valuable practical improvements; may explain much that was before obscure, and much, that, though successful, was but empirical; may distinguish partial from general affections of the nerves with more certainty, and enlarge the powers and applications of medicine. Diseases, of which the phenomena were apparently most arbitrary and intractable, must now become more clearly understood and more easily managed. Distinctions also may be more readily made between external phenomena depending on slight and temporary paralysis, occasioned by partial affections of the muscles of the face, and those more serious cases originating in the brain: and, of course, between affections of the symmetrical and the superadded nerves. The symptoms of what are really nervous disorders, affecting different systems, may be more confidently referred to the causes affecting what may be called their anatomical origins, and better arranged; and in very many forms of disorder, the signs which indicate danger, or afford reasonable ground of hope, signs connected with the respiratory system, will be more justly appreciated. Even those sudden and alarming occurrences will be less mysterious, in which instantaneous injury, or overwhelming pressure of the medulla oblongata, extinguishing the function of respiration at once, produces immediate death, those cases in which, as Dr. Bell observes, 'the change takes place with appalling suddenness; not a breath is drawn, nor a word uttered, nor a struggle to indicate pain, nor a feature discomposed.' And although this exact knowledge does not always confer immediate practical advantage, it seldom fails to lead to some ultimate benefit, and to the acquisition of some unforeseen resources against disease. Those who are engaged in the investigation of any science, can never see the full extent of the advantages which will reward their diligence, according to the sevenfold liberality with which intelligent industry is always repaid, that no inducement may be wanting to engage in it. The daily instances of respiration being affected in consequence of the presence of some offending matter in the stomach, so often, for instance, exemplified in asthma, are at once explained, when the close alliance between the stomach and respiratory nerves is clearly known; and the less distressing, but inconvenient affections of the same system, producing odd twitchings and catchings of the eyelids, or face, or shoulders, are no longer incomprehensible; and a nearer approach may be made towards understanding that singular disorder, called Angina Pectoris, which certainly does not always depend on a diseased state of the heart. Abundant pathological evidence is scattered through the pages of Mr. Bell's works, and bearing on all parts of the inquiry.

In one instance, the inconvenience of cutting a branch of the portio dura, going to the angle of the mouth, though not very great, was yet very marked; it was done by Mr. Bell himself, in taking out a tumour from before the ear of a coachman, and the man complained that he could no longer whistle to his horses. Numerous cases, indeed, of a similar kind, have now been amassed, and the Appendix to the Papers on the Nerves, consists entirely of such, chifly communicated to him by other practitioners, and completely proving all that is required.

